# Special Issue “Cisplatin in Cancer Therapy: Molecular Mechanisms of Action 3.0”

**DOI:** 10.3390/ijms24097917

**Published:** 2023-04-27

**Authors:** Valentina Gandin, James D. Hoeschele, Nicola Margiotta

**Affiliations:** 1 Department of Pharmaceutical and Pharmacological Sciences, Università degli Studi di Padova, 35131 Padua, Italy; valentina.gandin@unipd.it; 2 Department of Chemistry, Eastern Michigan University, Ypsilanti, MI 48197, USA; hoeschel@msu.edu; 3 Dipartimento di Chimica, Università degli Studi di Bari Aldo Moro, 70125 Bari, Italy

The year 2023 marks the 45th year since FDA approval of cisplatin as an anticancer drug, and, at present, it is widely used against a spectrum of human tumors, including early-stage ovarian cancer, non-small cell lung cancer (typically developed by smokers), head and neck, and advanced bladder cancer. Probably, the best results are obtained against testicular cancer, where cisplatin is 100% curative if the diagnosis of cancer is early. It has been estimated that 46% of patients undergoing chemotherapy in the clinics receive a platinum-based drug [1], and ca. 10.000 papers per year have been published in the period 2020–2022 (Scopus, March 2023) containing cisplatin in the title, abstract, or keywords. However, the mechanism of action of this drug is still not yet fully elucidated, although indisputable information has been obtained on the four key steps: (1) cellular uptake, (2) activation by aquation, (3) DNA binding, and (4) the processing of DNA lesions leading to cancer cell death. Evidence correlating the pharmacological effect of cisplatin with its capability to damage the structure of DNA is irrefutable, but the intimate connections between the causes and the effects (especially as relates to step 4) have not been fully demonstrated [2]. Complete explication of the mechanism of action of platinum-based drugs is still a fundamental and high-priority task that could significantly help medicinal inorganic chemists to rationally design new derivatives capable of ameliorating or eliminating the severe side effects that accompany patients’ treatments. Another important challenge is to understand in detail the nature of the intracellular pathways that are affected by platinum–DNA adducts, which are responsible for developing resistance and for the differential response of tumors to these platinum drugs (i.e., cisplatin and oxaliplatin have different activities toward colorectal cancer). This Special Issue aims to stimulate the study of the molecular determinants involved in the mechanisms of action of cisplatin and its analogs as an extremely important interdisciplinary field that requires the collaboration of chemists, biologists, pharmacologists, and physicians who, in some cases, do not always communicate on the same level. Considering that this is the 3rd Edition of the Special Issue, we think that the response from the scientific community has been satisfactory, with six papers and three reviews published. In particular, Sakamuro and associates investigated the neoplastic MYC transcription factor, which promotes malignant phenotypes of human cancers and increases resistance to cisplatin [3]. However, the signaling mechanisms through which MYC enhances resistance to cisplatin are not fully understood. In their study, two novel MYC effector pathways capable of cooperatively promoting resistance to cisplatin have been identified: (1) the PARP1-MYC pathway for BIN1 reduction, which spontaneously emerges when cancer cells become cisplatin-resistant, and (2) the ATM-phosphorylated MDC1-RNF8 signaling pathway produced by a BIN1 loss. From this investigation, it appears that clinically approved PARP and ATM inhibitors can be used in combination to re-establish the sensitivity to cisplatin in cisplatin-resistant cancer cells instead of directly targeting neoplastic MYC.

Wawruszak and associates conducted an isobolographic analysis and demonstrated that cisplatin and the histone deacetylase inhibitor cambinol possess an antagonistic effect in breast cancer cell lines with different phenotypes (estrogen-receptor-positive MCF7 and T47D and triple-negative MDA-MB-231and MDA-MB-468) [4]. Both agents, used separately, induced cell apoptosis, but, when used in combination in a fixed ratio of 1:1, they were able to enhance the antiproliferative effect in all the breast cancer cell lines used in the study, clearly indicating an antagonistic interaction. The evidence that came from the cell cycle analysis showed that cambinol was able to reactivate the cell cycle arrested in the S phase by cisplatin. Moreover, cell proliferation was increased by cambinol as compared to cisplatin used alone. The conclusion of this study is that combined chemotherapy with two or more agents is not always a convenient anticancer tool to obtain better therapeutic responses and preclinical investigations are necessary. In particular, against breast cancer, it is suggested that cambinol should be tested with chemotherapeutics that have different mechanisms of action other than CDDP.

Isobolographic analysis is also reported in the work of Stepulak and co-workers that investigated the influence of the Notch1 activity level on the pharmacological interaction between cisplatin and the histone deacetylase inhibitors valproic acid (VPA) and suberoylanilide hydroxamic acid (vorinostat) in luminal-like breast cancer cells [5]. Histone deacetylase inhibitors (HDIs) are emerging targets for cancer therapy and have been used to prepare Pt(IV) prodrugs, which display effective in vivo anticancer activity, possibly because of the synergistic effect of the intracellular release of cisplatin and of the HDI [6]. In the paper of Stepulak, the breast carcinoma MCF7 cells were genetically modified to express differential levels of Notch1 activity. The cytotoxicity of vorinostat or VPA, as determined through the use of an MTT assay, was higher in cells with decreased Notch1 activity and lower for cells with increased Notch1 activity than native MCF7 cells. The isobolographic analysis demonstrated that cisplatin and vorinostat or VPA, used in combination at a fixed ratio of 1:1, exerted additive or additive with a tendency toward synergism interactions. Therefore, in addition to Pt(IV) prodrugs combining cisplatin and HDIs in one molecule, combination therapy with cisplatin and HDIs at optimized ratios may be used against Notch1-altered luminal breast cancer. 

Since nephrotoxicity represents one of the major impediments of cisplatin chemotherapy, a deeper understanding of cisplatin-induced renal damage at the molecular level and the effect of potential protective agents is necessary. The search for successful nephroprotective agents and new biomarkers of renal damage and nephroprotection has led to cilastatin, which has proved, in vitro and in vivo, to exert a nephroprotective effect in cisplatin therapies, recently entering clinical trials. Moreno-Gordaliza and co-workers have used a targeted lipidomics approach, using LC-MS/MS, for the quantification of 108 lipid species (including phospholipids, sphingolipids, and free and esterified cholesterol) in kidney cortex and medulla extracts from rats treated with cisplatin and/or cilastatin [7]. The results have shown, after cisplatin treatment, an alteration in the cortex and medulla up to 56 and 63 lipid species, respectively. Contrarily, co-treatment with cilastatin attenuated many of these lipid alterations, either totally or partially, with respect to control levels. Multivariate analysis revealed that lipids can be used to discriminate renal damage and nephroprotection and that cholesterol esters are the most discriminating species, as well as sulfatides and phospholipids. Finally, potential diagnostic biomarkers of cisplatin-induced acute kidney damage and cilastatin nephroprotection were also identified in this investigation.

Adar and colleagues investigated the mechanisms responsible for the acquired resistance shown by the resistant phenotype of the A2780 ovarian cancer cell line (A2780cis) by conducting a comprehensive genomic characterization of sensitive and resistant A2780 cell lines [8]. Since cisplatin kills cancer cells by damaging their DNA, the authors measured DNA damage and found that A2780cis acquired less damage, but not because of a faster repair, as the overall NER efficiency was similar in both resistant and sensitive cells. The genome-wide mapping of NER showed a shift in the resistant cells from a global genome toward the transcription-coupled repair. Moreover, by mapping the gene expression changes following cisplatin treatment, 56 upregulated genes that have a higher basal expression in A2780cis were identified, suggesting they are primed for a cisplatin response in the resistant cell line. Although many of these primed genes have been previously recognized in cisplatin or DNA damage response, most have not yet been characterized. Six out of the seven primed genes investigated (ARC, EGR2, MYLIP, OSGIN1, RHOV, TBR1, and SNAI1) were upregulated in response to cisplatin in three additional cell lines tested in this study (293T, transformed human embryonic kidney cells; A549, lung adenocarcinoma cancer; and U2OS, bone osterosarcoma), making them attractive candidates for further investigation since they could be important prognostic markers or targets for tailored combined therapy in the future.

Many research groups operating in the field of antitumor platinum-based compounds have dedicated efforts to the development of platinum (IV) prodrugs. Pt(IV) prodrugs have been designed to overcome the problems generally associated with traditional platinum(II) chemotherapy, such as side effects and the development of chemoresistance, which partially limit the efficacy of this family of drugs [9,10]. Recent work reported an investigation of the Pt(IV) prodrug kiteplatin ([PtCl_2_(*cis*-1,4-DACH); DACH = diaminocyclohexane), which has two benzoate ligands coordinated in the axial positions: *cis,trans,cis*-[PtCl_2_(OBz)_2_(*cis*-1,4-DACH)] (OBz = benzoate) [11] This compound, similar to other Pt(IV)-benzoate derivatives reported by other groups [12], showed cytotoxic activity at a nanomolar concentration. The investigation has been extended to the in vivo activity of *cis,trans,cis*-[PtCl_2_(OBz)_2_(*cis*-1,4-DACH)] in a Lewis Lung Carcinoma (LLC) model and its suitability for oral administration. The kiteplatin–benzoate prodrug resulted in being remarkably stable at pH 1.5 (as that found in the stomach environment); thus, the results indicated that it is amenable for oral administration. Interestingly, in the in vivo LLC model, a comparable reduction in tumor mass (~75%) was observed after administering *cis,trans,cis*-[PtCl_2_(OBz)_2_(*cis*-1,4-DACH)] through oral gavage, indeed indicating that there is the possibility of oral administration. 

Pt(IV) prodrugs are also the object of the review published in the Special Issue by Spector, Krasnovskaya and colleagues [13]. In particular, Pt (IV) prodrugs with non-steroidal anti-inflammatory drugs (NSAIDs) as axial ligands, published in the past seven years, have been reviewed. This interest stems from the fact that a chemo–anti-inflammatory strategy is of interest for the treatment of aggressive cancers, which have often been associated with inflammatory processes [14]. Generally, NSAIDs are lipophilic ligands that, once coordinated to Pt (IV) compounds, enable efficient entry into tumor cells and are reduced through intracellular reductants to release cytotoxic Pt(II) species and NSAIDs, thereby reducing the side effects and increasing the therapeutic efficacy of platinum chemotherapy. In addition to summarizing the studies devoted to the development of Pt (IV) prodrugs with NSAIDs as axial ligands, the review reports also the investigations on their mechanism of action, anti-inflammatory activity, structure–activity relationships, and therapeutic efficacy.

The review by Bandiera Paiva and associates provided a summary of the investigations published under the mechanistic rationale of combined intratumor injections of cisplatin and laser-induced thermal therapy (CDDP–LITT) and the clinical application of such minimally invasive treatment for cancer [15]. It has been demonstrated that the antitumor potential of cisplatin can be potentiated by hyperthermia compared to drug treatment alone. Powerful and monochromatizable radiations that can induce an Auger electron cascade in cisplatin molecules have been recently obtained by accelerators of high-energy particles, such as synchrotrons, rendering the photoactivation of cisplatin theoretically possible. Both heat and light increase cisplatin anticancer activity via multiple mechanisms, and, in the past 27 years, the authors of the review have developed infrared photo-thermal activation of cisplatin for cancer treatment, and future aims include the development of photoactivatable Pt-prodrugs to be injected intratumorally to increase the selectivity, lower the systemic toxicity, and increase the efficacy of Pt-based drugs. This approach could be particularly important in the treatment of tumors that are accessible to laser-based fiber-optic devices, such as those of the head and neck.

In the third review published in this Special Issue, Ali, Ramotar and colleagues raise a Hamletic question about the possibility of improving the therapy with platinum-based drugs using new non-genomic targets [16]. So far, several preclinical and clinical studies have struggled to overcome the major limitations associated with antitumor platinum-based chemotherapy, but no definitive solution has been found. A more comprehensive molecular and genetic profiling of patients appears to be necessary to identify those patients that can really benefit from cisplatin and its analogs. Moreover, cisplatin treatment regimens can be improved if the drug is administered in combination with additional targeted therapies to achieve an ideal equilibrium between the toxicity toward the tumor and its tolerance mechanisms. In this review, the authors discuss the importance of the different biological mechanisms responsible for the acquired resistance of some tumors to cisplatin and its analogs, highlighting the processes that can be modulated to suppress cisplatin resistance and providing an insight into the role of uptake transporters in enhancing drug efficacy.

Finally, as Guest Editors, we would like to thank all of the contributors, peer reviewers, editors, and MDPI’s publishing team for their efforts in publishing this Special Issue, “Cisplatin in Cancer Therapy: Molecular Mechanisms of Action 3.0”.
